# Sex-Specific Differences in Shoaling Affect Parasite Transmission in Guppies

**DOI:** 10.1371/journal.pone.0013285

**Published:** 2010-10-11

**Authors:** E. Loys Richards, Cock van Oosterhout, Joanne Cable

**Affiliations:** 1 School of Biosciences, Cardiff University, Cardiff, United Kingdom; 2 Evolutionary Biology Group, University of Hull, Hull, United Kingdom; University of Alabama, United States of America

## Abstract

**Background:**

Individuals have to trade-off the costs and benefits of group membership during shoaling behaviour. Shoaling can increase the risk of parasite transmission, but this cost has rarely been quantified experimentally. Guppies (*Poecilia reticulata*) are a model system for behavioural studies, and they are commonly infected by gyrodactylid parasites, notorious fish pathogens that are directly transmitted between guppy hosts.

**Methodology/Principal Findings:**

Parasite transmission in single sex shoals of male and female guppies were observed using an experimental infection of *Gyrodactylus turnbulli*. Parasite transmission was affected by sex-specific differences in host behaviour, and significantly more parasites were transmitted when fish had more frequent and more prolonged contact with each other. Females shoaled significantly more than males and had a four times higher risk to contract an infection.

**Conclusions/Significance:**

Intersexual differences in host behaviours such as shoaling are driven by differences in natural and sexual selection experienced by both sexes. Here we show that the potential benefits of an increased shoaling tendency are traded off against increased risks of contracting an infectious parasite in a group-living species.

## Introduction

Social aggregation of fish, or shoaling behaviour, involves individuals trading-off the costs and benefits of group membership [1]. Shoaling is thought to have evolved as an antipredator response [2], but also provides foraging benefits as individuals in shoals can allocate more time to feeding [3] and profit from improved food location [4]. Benefits are maximised with increasing shoal size through, for example, improved vigilance and attack dilution (e.g., [5]), as well as increasing levels of coordination and phenotypic homogeneity within the group [6]. However, competition for resources also increases with group size [7], and a number of studies have documented an increase in risk-taking behaviour with increased shoal size (e.g., [3]).

Parasitism may impose yet another cost of group living, since for uninfected individuals the probability of acquiring a directly transmitted parasite increases with the formation of a group [8], [9]. Indeed, there is strong evidence of a positive correlation between host group size and parasite prevalence and load [10], [11]. Many parasites have been assessed for their impact on fish behaviour (reviewed in [9]) but only a limited number of studies have considered the reverse situation, i.e. the direct impact of shoaling behaviour on parasite transmission [12], [13], [14]. This latter study [14] found that for some groups of parasites, shoaling host species harboured significantly greater parasite diversity than solitary host species, but this pattern did not hold for directly transmitted parasites. It is important to assess the impact of host shoaling behaviour on this class of parasite since they are likely to be greatly influenced by the shoaling behaviour of their hosts, as transmission can occur directly between shoal or group members. A ubiquitous and highly contagious group of fish parasites are the gyrodactylid monogeneans. They are directly transmitted but the possible influence of host shoaling behaviour on their transmission has never been examined.

A particularly well studied host-parasite system is the guppy (*Poecilia reticulata*) and its infectious parasite, *Gyrodactylus turnbulli*. Guppies are small tropical fish and an important ecological and evolutionary model. They have been widely used to explore host adaptations to natural and sexual selection pressures (e.g., [15], [16], [17]). Guppies are sexually dimorphic, with males being more colourful and smaller than females, and there are marked behavioural differences between the sexes [18]. They breed throughout the year and males spend a large proportion of time in courtship displays and sneaky mating attempts. Female guppies shoal more than males and show a greater preference for associating with their familiar shoal mates [19]. Furthermore, guppies living in a high predation area shoal significantly more than fish in low predation streams and this may facilitate interhost transmission of ectoparasites [20].

In natural guppy populations, gyrodactylids are the most prevalent parasitic worms [21]. Amongst wild caught Trinidadian guppies, the parasite load of *Gyrodactylus* spp. is generally less than 10 worms/host [22], but can be as high as 100 parasites [23]. Larger guppies tend to harbour more parasites [24], and the maximum parasite load increases exponentially with increased host body size [25]. These parasites give birth to a fully-grown offspring, which attaches to the host alongside its parent and already contains a developing embryo (reviewed in [26]). They also have a short generation time of just 24 h (at 25°C) [27] resulting in rapid population growth. Gyrodactylids can be an important selective force in natural guppy populations [28] and they adversely affect male colouration in guppies [29]. Infection also causes a number of behavioural changes in the host, such as a reduced feeding rate [30] and a reduced ability to compete for food with uninfected conspecifics [31]. Also, fish with heavy infections can develop clamped fins [32] which presumably reduces swimming performance.

Here, we investigate whether the degree of host contact in single-sex shoals influences gyrodactylid transmission, and whether guppy behaviour is influenced by the presence of infected conspecifics.

## Materials and Methods

### Host and parasite origins

Ornamental (petshop) guppies (n = 108) were purchased from a UK commercial supplier. On arrival at Cardiff University all fish were briefly anaesthetised in 0.02% MS222 and externally screened for visible parasites under a stereo-microscope with fibre optic illumination. All fish were infected with Gyrodactylus spp. but were subsequently treated with 0.2% levamisole (Norbrook, UK) and screened clear for visible parasites at least three times (see [33]) and then left for three to four months before use. The guppies were maintained under a 12 h light: 12 h dark lighting regime in mixed-sex groups (1∶5 male to female ratio) with about 30 fish per aquaria (45×45×120 cm), and fed on a diet of flakes (Aquarian®) and frozen bloodworm. An isogenic strain of Gyrodactylus turnbulli (strain Gt3), originally isolated from ornamental guppies in 1997, was used for all infections. All experiments were conducted at 25±1°C.

### Experimental design

Single sex groups of male or female guppies (6 individuals per group) were placed in test tanks (60×30×40 cm), and allowed to acclimate for 5 d. Standard length was controlled by size matching all individuals within a tank and by only using fish within a 20–30 mm size range. Male guppies did not have very large fan or forked tails. Each aquarium contained an air supply and water filter. The location of male and female guppy tanks was randomised, and guppies in different aquaria were visually and physically isolated from one another. A single guppy in each tank was randomly assigned as the focal fish. This focal guppy was recognisable by its colour pattern (in males) or pigment patch on the caudal fin (in females). The experiment was conducted in three separate batches for logistical reasons.

After acclimation, the shoaling behaviour of each group was observed once daily for 3 consecutive days (t = Days 1–3). All observation periods lasted 15 min per group (5 min in total for each shoaling behaviour parameter). During each observation period, 10 measurements of nearest neighbour distance were made for each focal fish, and for one, randomly chosen, non-focal fish per tank. A further 10 measurements of shoal size were recorded, by counting the number of fish in the largest shoal at the time of observation. The time interval between each of these measures was 30 s, which was sufficient to make consecutive observations independent. Also the time spent shoaling by both focal and non-focal fish was measured over 5 min. Horizontal and vertical lines drawn every 2 cm on three sides (back and two sides) of each test aquaria facilitated the estimation of between-individual distances, as all shoaling behaviour measurements were evaluated in three-dimensional space. Shoal members were defined as fish within 4 body lengths of one another [1].

At the end of Day 3, all fish were removed from the test aquaria and kept individually in one litre containers (to prevent restructuring of social groups) while the focal fish from each test tank was infected with *G. turnbulli*. Infection was achieved by anaesthetising each focal individual and allowing them to contact a euthanized heavily infected same-sex fish (donor) in a watch glass containing 0.02% MS222 on the stage of a stereo-microscope. The focal fish was removed once ca. 100 worms had transferred from the donor. Success of parasite transfer was estimated after 24 h by confining each focal fish in a crystallizing dish (5 cm diameter) containing dechlorinated water on the stage of a stereo-microscope and counting the number of parasites under fibre optic illumination. Non-focal fish were sham infected under anaesthetic using a similar procedure. Following infection, all fish were returned to their test tank (t = Day 4). There was no evidence of secondary pathology (such as clamped fins or reduced mobility) among focal fish at this time.

Shoaling behaviour was again measured for 3 consecutive days following infection. On the first day post-infection (t = Day 4), observations were made twice: once in the morning at 10:00 (1 h after the focal fish were returned to their home tank) and once at 14:00. During Days 5–6, observations were made once daily.

Trials (one male tank and one female tank) were repeated 9 times (18 tanks in total) and no fish was tested more than once. At the end of each trial the extent of within-shoal parasite transmission was assessed by recording the number and position of parasites on each individual fish anaesthetised in 0.02% MS222. No fish deaths occurred during the experiment and no fish presented with clamped fins (pathology characteristic of *G. turnbulli* infections) on Day 6.

### Data analysis

The data from all the trials was pooled and analysed to test whether within-shoal parasite transmission was dependent on an individual's behaviour, sex or density of parasite load. A preliminary analysis showed that the parasite loads and shoaling parameters were not normally distributed, and therefore the data was natural log-transformed. This resulted in normality of residuals, established using Anderson-Darling tests. Furthermore, preliminary analysis showed that there were no significant differences in fish shoaling behaviour, parasite growth or transmission between trials (‘Tanks’) and batches (‘Batch’) of the experiment. A Repeated Measures ANOVA was used to analyse whether differences in the three parameters of shoaling behaviour were explained by the day of the experiment, sex and infection status of the guppy. Day of experiment (‘Day’) was used as a covariate and infection status (‘Parasitised’) was crossed with sex (‘Sex’) as factors.

All guppies were assessed for parasite burdens at the end of the 3-day infection period. A parasite burden is defined as the total number of parasites per fish host. Differences in initial and final parasite burdens were assessed using Kruskal-Wallis tests. Comparisons between males and females in their ability to spread infection to conspecifics were tested using χ^2^ analysis, with the standard errors calculated using jackknife analysis. This was done by comparing the numbers of male and female non-focal fish that were carrying a naturally acquired gyrodactylid infection at the end of the experimental period, distinguishing four categories: (i) males that carried a parasite burden (‘male infected’), (ii) uninfected males (‘male clean’), (iii) females that carried a parasite burden (‘female infected’) and (iv) uninfected females (‘female clean’). Focal fish were excluded from this analysis, as these fish were experimentally infected.

A binary logistic regression analysis (logit) was used with a dichotomous dependent variable, infected or not infected (coded as ‘1’ and ‘0,’ respectively), to test whether the infection status of fish at the end of the experimental period was associated with initial parasite load of focal fish (‘Gyrostart’) and sex (‘Sex’) of the guppy. The model uses ‘Sex’ as a fixed factor crossed with ‘Gyrostart’ as covariate. We used an iterative re-weighted least squares algorithm to obtain maximum-likelihood estimates of all parameters. The log-likelihood was used to test whether the coefficients of the predictors were significantly different from zero. A logit link function was employed to calculate the odds ratio and its 95% confidence interval (CI). The odds ratio represents the ratio in which an event occurs relative to a reference event. All statistical analyses were performed using Minitab 15.

## Results

As predicted from Griffiths and Magurran [19], female guppies shoaled significantly more than males. This resulted in focal females passing on their infection to non-focal conspecifics more readily than focal males in single sex shoals.

### Shoaling behaviour

**Figure 1:**
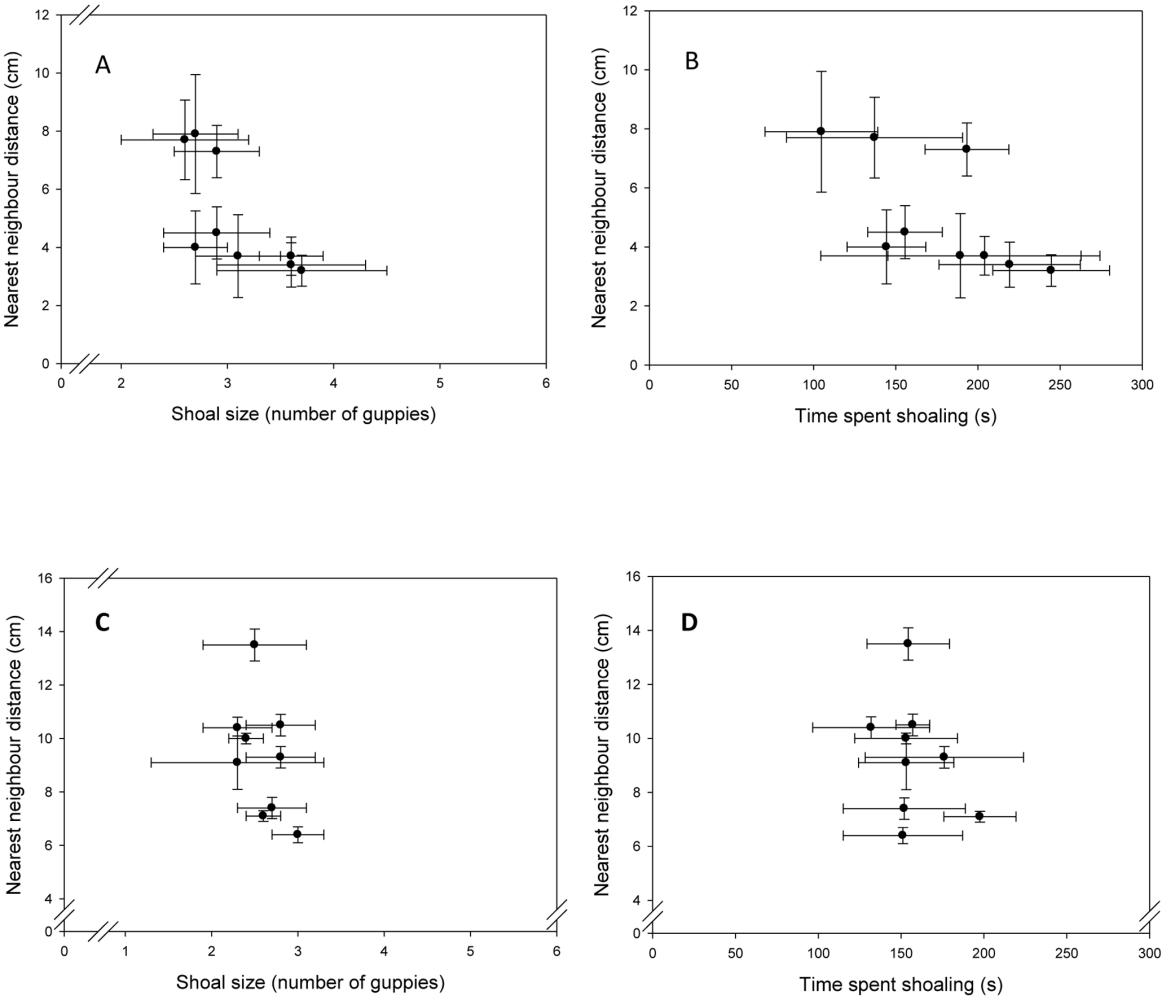
Correlation between shoaling behaviours separated by sex. Correlation between the nearest neighbour distance and (A) the number of guppies per shoal, and (B) the time spent shoaling for female guppies. (C) and (D) represent the same correlations respectively for male guppies. Shown are the mean and standard deviation.

First we analysed the correlation between our three shoaling parameters (shoal size, average distance between nearest neighbours and the duration shoaling) separately for male and females guppies. These parameters are strongly correlated to one another for female fish but not for males (Pearson's correlation analyses: females all r≥0.343, all P≤0.006, males all r≤0.152, all P≥0.235, Figures 1A–D).

**Figure 2:**
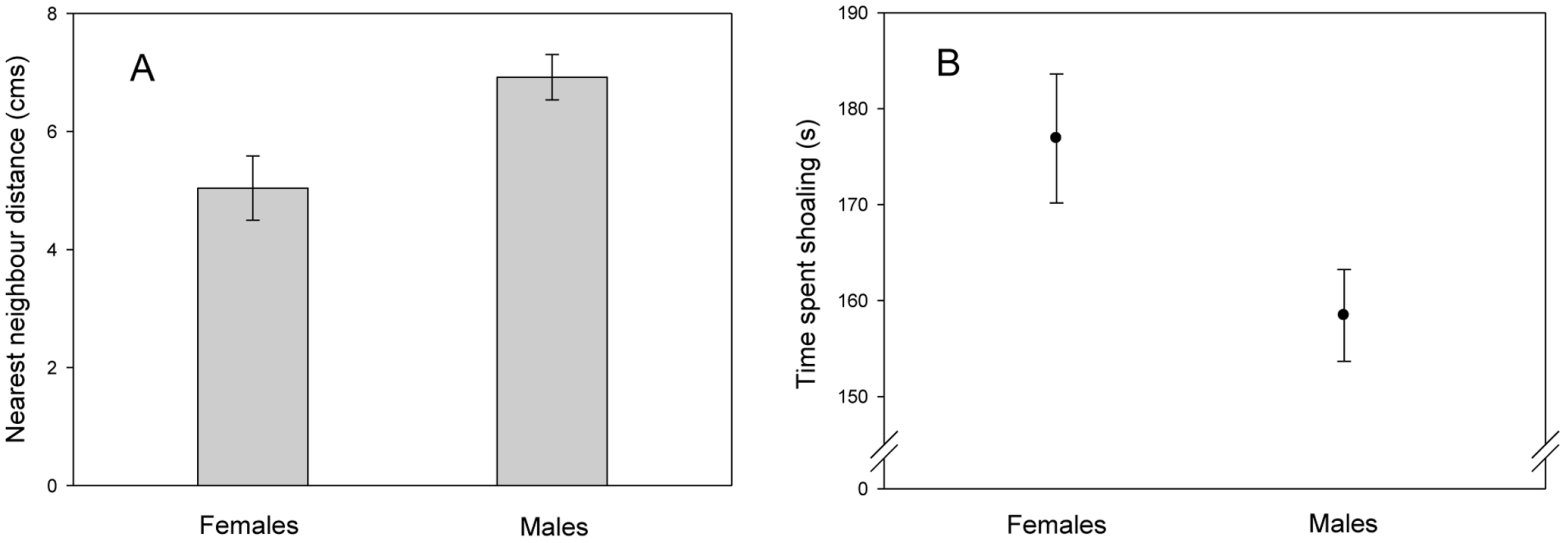
Female guppies exhibit increased shoaling behaviour compared to males. (A) Mean ±SE nearest neighbour distance of non-focal female and male guppies, pooled for Days 1–6. (B) Mean ±SE time spent shoaling by non-focal female and male guppies pooled for Days 1–6.

Female guppies formed larger shoals than males with a significantly higher ‘average shoal size’ (Repeated measures ANOVA: F_1,121_ = 25.16, P<0.001), and both focal and non-focal females formed significantly tighter shoals separated by shorter ‘nearest neighbour distances’ compared to males (Focal fish: F_1,103_ = 6.47, P = 0.012; Non-focal fish: F_1,121_ = 25.28, P<0.001) (Figure 2A). Focal female guppies spent an average of 155.3±8.3 s per 5 min shoaling compared to 141.0±8.1 s for focal males, although this difference was not significant (F_1,121_ = 1.68, P = 0.099). Finally, non-focal females spent significantly longer shoaling with conspecifics than non-focal males (F_1,121_ = 4.38, P = 0.038) (Figure 2B).

### The effects of parasites on shoaling

**Figure 3:**
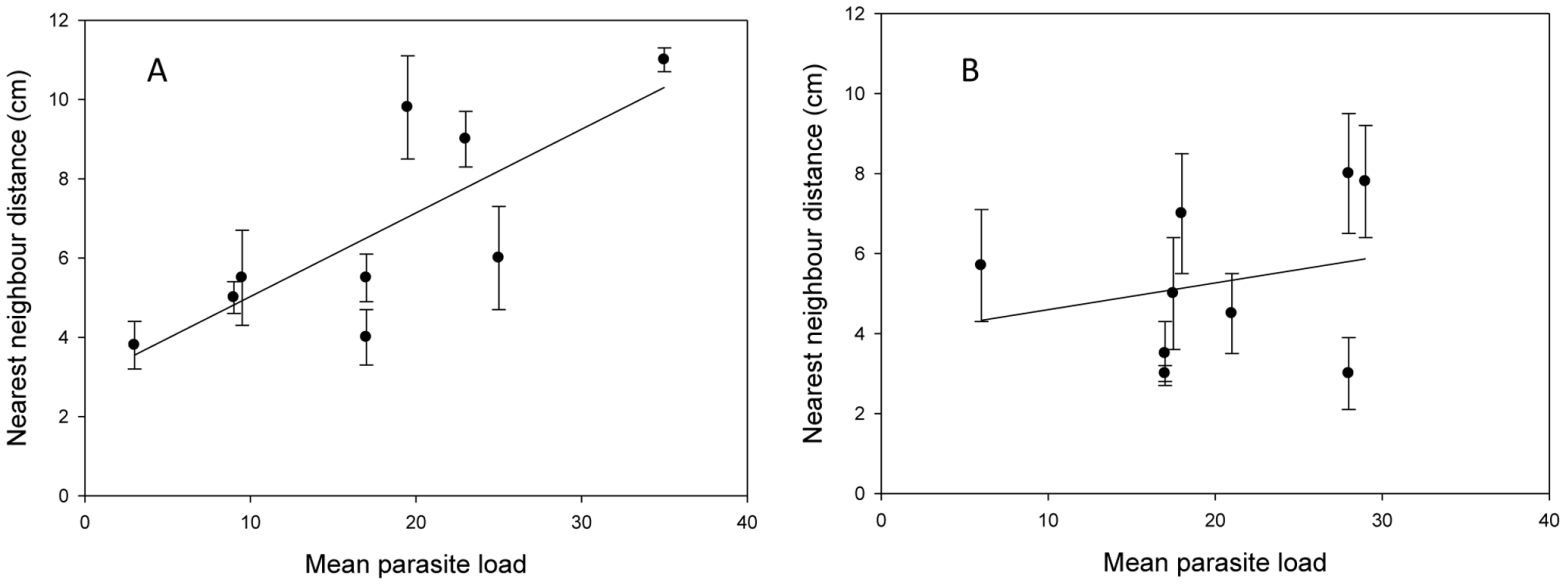
Relationship between shoaling behaviour and parasite load. Nearest neighbour distance and parasite load for female (A) and male (B) guppies. Shown are the mean and standard deviation for both nearest neighbour distance and parasite load, averaged across the individuals within a tank.

Surprisingly, there were only marginal effects of parasitism on the shoaling behaviour in guppies. Neither mean shoal size (Pearson's correlation: r = 0.190, P = 0.450), nor the duration shoaling (Pearson's correlation: r = 0.427, P = 0.077) was affected by the average number of parasites per guppy in the tank. A significant positive correlation was detected between the average distance between nearest neighbours and mean parasite load for female guppies (Regression: F_1,7_ = 9.27, P = 0.019) but not for males (F_1,7_ = 0.56, P = 0.480) (Figure 3A and B) (Pearson's correlation: r = 0.506, P = 0.032). This shows that with an increased number of parasites, female guppies remain on average at a larger distance from one another.

### Parasite transmission

**Figure 4:**
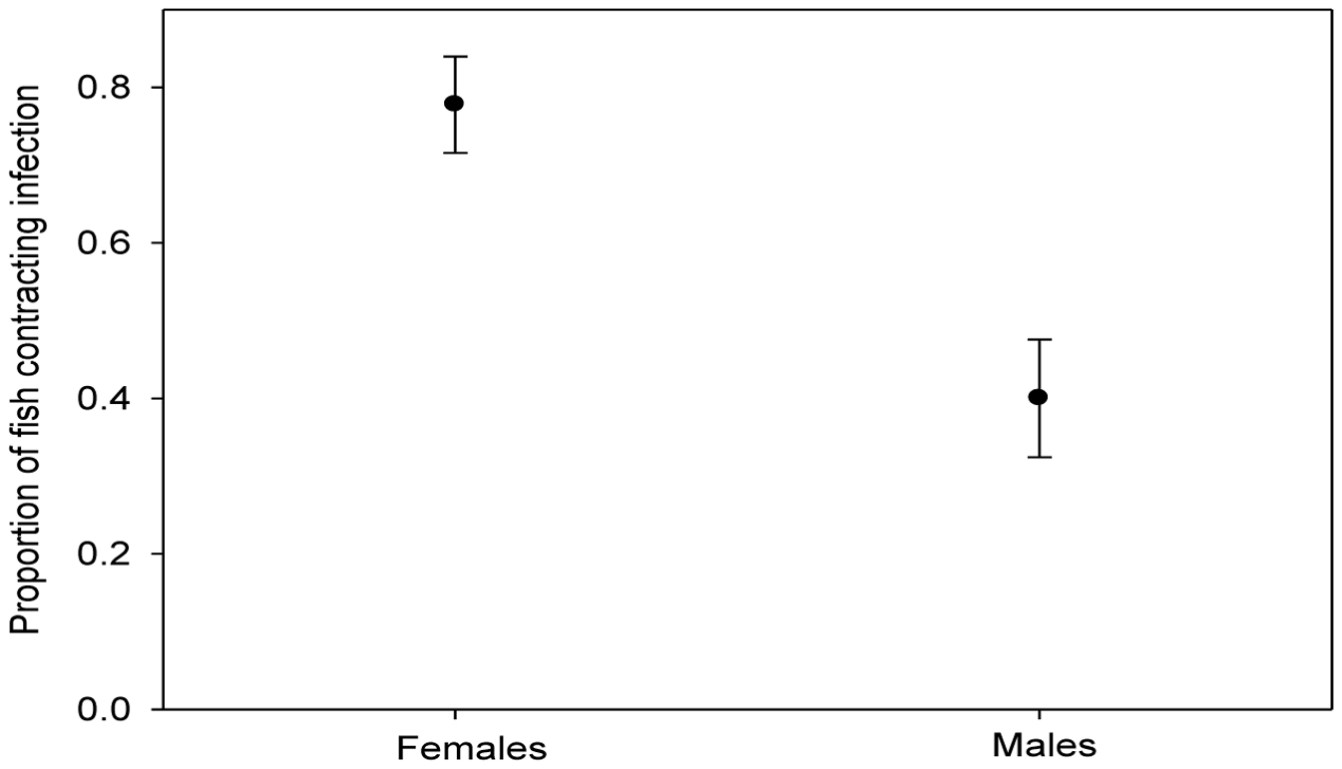
Proportion (mean ±SE) of non-focal male and female guppies contracting a *Gyrodactylus turnbulli* infection.

There was no significant difference between initial parasite loads of focal males (mean load: 104±18.1 worms/fish) and females (117±16.1 worms/fish) (Kruskal-Wallis: H = 0.34, DF = 1, P = 0.562). During the infection period, there was significantly higher parasite population growth on male focal fish compared to females (Kruskal-Wallis: H = 5.48, DF = 1, P = 0.019). At the end of the infection period, there were significantly more non-focal females infected than non-focal males (χ^2^ = 13.264, DF = 1, P<0.001) (Table 1). Indeed, non-focal female guppies were four times more likely to become infected than non-focal males (Binary logistic regression: Z = 2.46, P = 0.014: Mean (5–95% CI) Odds ratio = 4.33 (1.35–13.92)) (Table 2; Figure 4). This indicates that parasite transfer was more efficient between female guppies (female to female) than between males (male to male).

**Table 1:** Contingency table with counts of clean (non-infected) and infected non-focal female and male guppies, at the end of the 3-day infection period.

	Females	Males	Total
Clean	10	27	37
Infected	35	18	53
Total	45	45	90

**Table 2:** Binary logistic regression for infection status (0 - clean, 1 – infected) with sex of the host (‘Sex’) as a factor crossed with the initial tank burden (‘GyroStart’) as covariate.

Predictor	Coef	StDev	Z	P	Mean Odds Ratio	Odds Ratio5–95% CL
Constant	−0.1220	0.4668	−0.26	0.794	*	*
Sex	1.4667	0.5954	2.46	0.014	4.33	1.35–13.92
GyroStart	0.0006	0.0019	0.32	0.747	1.00	1.00–1.00
Sex × GyroStart	0.0029	0.0043	0.70	0.486	1.00	0.99–1.01

## Discussion

There was a significant difference between the sexes in the degree of contact within single sex shoals of male and female guppies. Two of the three measures of shoaling behaviour (distance to nearest neighbour and time spent shoaling) used in the current study indicated that females shoaled significantly more than males. We believe that this sex-specific difference in shoaling behaviour explains why females were four times more likely to become infected with the ectoparasite, *Gyrodactylus turnbulli* than males. Males, on the other hand, sustained parasites with the fastest population growth rate, possibly because compared to females, they were less likely to lose parasites by transmission to conspecifics.

Contrary to our expectations, non-focal individuals did not alter their shoaling behaviour following introduction of the infected focal fish. Previous studies have shown that fish exhibit aversion behaviour to limit contact with parasites and avoid joining shoals that contain parasitised members (e.g., [34], [35]). Recently, Tobler and Schlupp [36] provided evidence that both parasitised and unparasitised cave mollies (*Gambusia affinis*) prefer to shoal with uninfected conspecifics. Fish are also known to avoid particular types of habitat associated with infection risk (e.g., [37]) as well as rejecting infected sexual partners (e.g., [38]). Parasite-mediated selection can be much reduced in captivity [39], [40], and hence, the ornamental (petshop) guppies used in the current study may have lost the appropriate aversion response to infected conspecifics common in many wild fish species (reviewed in [9]). The guppies used in our experiment may have been in captivity for as long as 300 generations [39], and as a result, they could have lost the appropriate behavioural response to parasite infection. We believe that this crucial difference can explain the disparity between our results and previous studies on the effects of parasitism on shoaling.

The elevated rate of parasite transfer in females appeared to be due to increased host contact rather than faster parasite population growth rate [24], [41]. Sex-specific differences in shoaling [19] resulted in more host contact between females, which increased female-to-female parasite transmission. Of course, other factors influencing parasite transmission rate within fish shoals need to be considered. Fish populations may differ in predation risk, mating and/or foraging behaviour, which in turn can affect shoaling behaviour and thereby parasite transmission (e.g., [42], [31]). For example, male guppies are known to have a lower propensity to shoal compared to females, instead preferring to move between shoals of female guppies searching for mating opportunities [43]. Male behaviour could transfer parasites between shoals of females in a single pool or section of stream. Also, male behaviour may vary between different guppy populations, which could result in differences in parasite transmission between populations. Parasite transmission may also be linked with differences between wild and captive-bred fish, a point made previously but equally valid here. For example, van Oosterhout et al. [40] found dramatic differences in parasite load between wild and captive-bred fish when they occurred in the same (semi-natural) conditions in Trinidad, with 94% of captive-bred fish carrying an infection compared to only 40% of individuals in a wild population.

In summary, we have shown the impact of sex-specific differences in shoaling behaviour on parasite transmission within a group-living host species. We show that females have a higher tendency to shoal than males, and importantly, quantify a fitness-cost of shoaling. We demonstrate that guppies are exposed to a considerable risk of contracting a gyrodactylid infection by shoaling with parasitised conspecifics. We hope in future to more clearly separate the influence of sex and host behaviour on the spread of parasites within a host group, and explicitly test the relationship between shoaling and parasite transmission.
